# Artificial nucleic acid backbones and their applications in therapeutics, synthetic biology and biotechnology

**DOI:** 10.1042/ETLS20210169

**Published:** 2021-07-23

**Authors:** Sven Epple, Afaf H. El-Sagheer, Tom Brown

**Affiliations:** 1 Chemistry Research Laboratory, University of Oxford, Oxford OX1 3TA, U.K.; 2 Chemistry Branch, Department of Science and Mathematics, Faculty of Petroleum and Mining Engineering, Suez University, Suez 43721, Egypt

**Keywords:** chemical modification, oligonucleotides, synthetic biology, therapeutics

## Abstract

The modification of DNA or RNA backbones is an emerging technology for therapeutic oligonucleotides, synthetic biology and biotechnology. Despite a plethora of reported artificial backbones, their vast potential is not fully utilised. Limited synthetic accessibility remains a major bottleneck for the wider application of backbone-modified oligonucleotides. Thus, a variety of readily accessible artificial backbones and robust methods for their introduction into oligonucleotides are urgently needed to utilise their full potential in therapeutics, synthetic biology and biotechnology.

Chemical modification of DNA and RNA play myriad roles in therapeutics, diagnostics and synthetic biology. Oligonucleotides (ONs) can be modified at the nucleobase, the sugar, or the phosphodiester (PO) backbone (Figure 1) [1,2]. Backbone modifications can generate remarkably biomimetic constructs such as therapeutic oligonucleotides (TherONs) [2], xenobiotic genetic polymers [3–5], aptamers [4,6–8], ribozymes [9], single guide RNAs [10] and synthetic genes [11–13]. Many artificial backbones are known, but their applications in therapeutics and synthetic biology remain limited. This perspective focuses on artificial nucleic acid backbones and briefly describes the most common strategies for their synthesis. Selected examples demonstrate their potential and highlight the current limitations of this technology.

**Figure 1.**
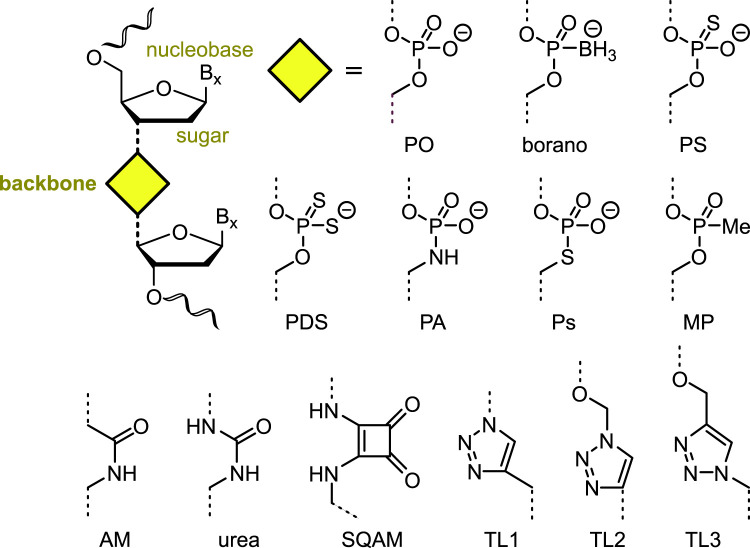
Selected backbone modifications. The nucleobase (B_x_), sugar and backbone are labelled in the dinucleoside structure. Backbone modifications are represented by a yellow diamond.

## Therapeutic oligonucleotides

### Synthesis

**Figure 2.**
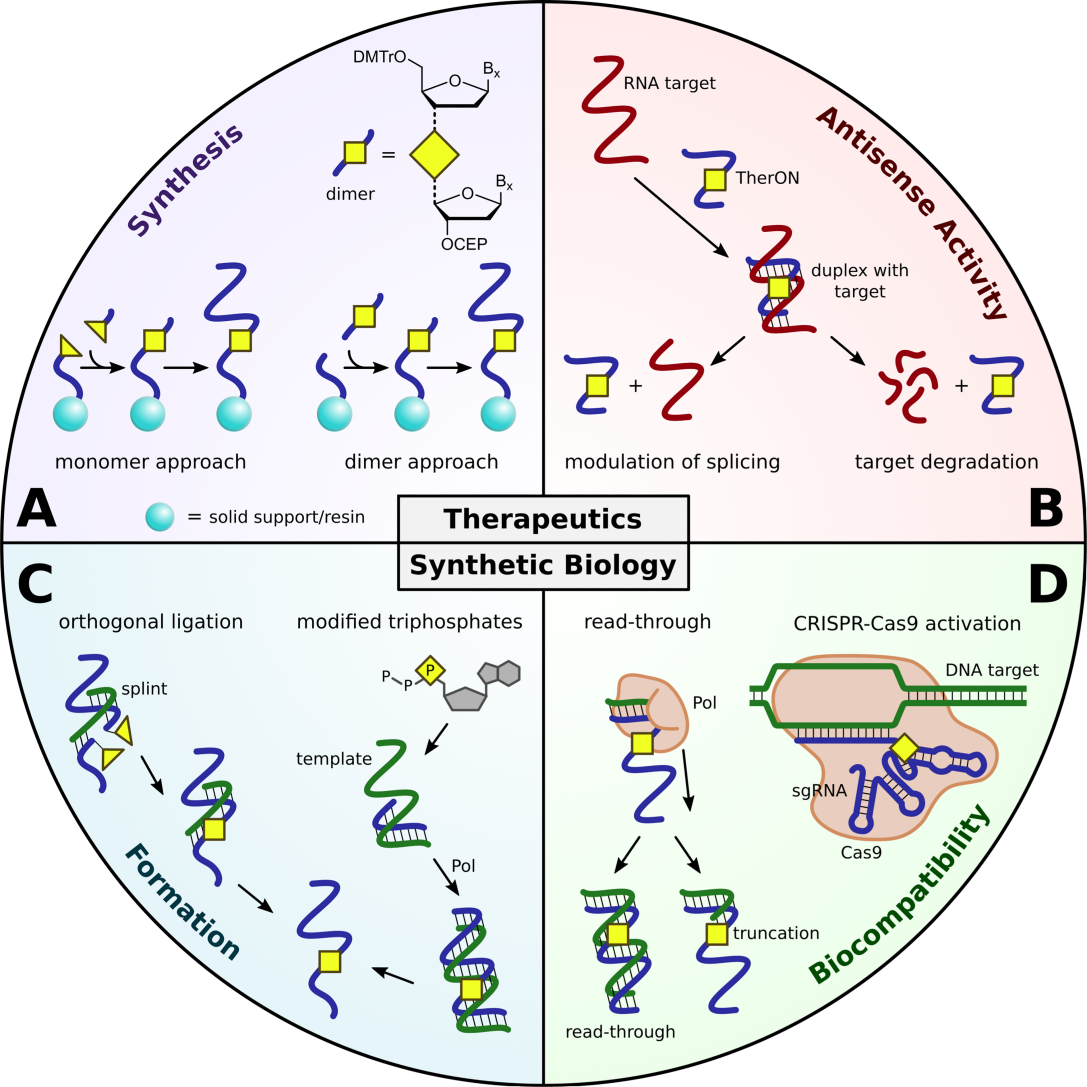
Modified backbones for applications in therapeutics and synthetic biology. Applications of artificial backbones can be divided into therapeutics and synthetic biology (top and bottom half of the circle, respectively). Yellow triangles represent reactive functional groups and yellow squares represent artificial backbones. (**A**) Synthetic approaches for backbone modified TherONs. Monomer approach: Functional groups react to form the artificial backbone during ON synthesis on a solid support. Dimer approach: the artificial backbone is part of a 4,4′-dimethoxytrityl (DMTr)-protected dinucleoside cyanoethyl phosphoramidite (CEP) that can be coupled using standard ON synthesis conditions. (**B**) Antisense activity of a chemically modified TherON. Hybridisation of the TherON with a target RNA can either lead to alternative splicing or degradation of the target RNA. (**C**) Strategies to access long chemically modified ONs for applications in synthetic biology. Orthogonal ligation: Functional groups of short ONs react to form the artificial backbone. This ligation is often facilitated by a splint (template) ON. Modified triphosphates: the artificial backbone can be part of a modified triphosphate that is a substrate for a polymerase (Pol). Incorporation of the modified triphosphate leads to sites with artificial backbones. (**D**) Selected examples of long backbone modified ONs in synthetic biology. Polymerase read-through: Compatible artificial backbones in genetic templates can be read by polymerases to produce a replicon with the complementary sequence. Incompatible artificial backbones lead to truncation or mutation sites during replication. CRISPR–Cas9 activation: Backbone modified sgRNAs can direct Cas9 to sequence-specific sites in DNA to facilitate cutting of the DNA target.

The preparation of TherONs, which are typically short (∼20mer) chemically modified ONs [14,15], mainly relies on solid support-based oligonucleotide synthesis [16,17]. Artificial backbones are usually introduced by modified on-resin coupling of a monomer to form the unnatural backbone (monomer approach, Figure 2A), or by coupling of a dinucleoside containing the artificial linkage (dimer approach, Figure 2A). The monomer approach presents significant challenges due to the potential chemical incompatibility with standard ON chemistry and synthesis equipment. Nevertheless, on-resin formation of artificial backbones has been reported for boranophosphates (borano) [18], phosphorothioates (PS) [19], phosphorodithioates (PDS) [20], phosphoramidates (PA) [21], methylphosphonates (MP) [22,23], amides (AM) [24–27] and to a limited extend for triazole linkages (TLs) [28]. Indeed, the dimer approach is preferred for backbones that are harder to form on a solid support, such as ureas [29], squaramides (SQAM) [30], or triazoles [31–36]. The artificial linkage can be pre-formed as part of a dinucleoside that is compatible with standard oligonucleotide synthesis. However, 16 modified dinucleosides are required to cover all sequence possibilities and the construction of consecutive artificial backbones is not possible. In general, the limited availability of optimised and easy-to-use protocols for on-resin formation of artificial backbones, and the demands of the dimer approach remain major bottlenecks in research in the TherON area.

### Antisense activity

From the plethora of artificial backbones, only a limited number of chemical modifications are found in clinically approved TherONs [37]. Synthetic accessibility, efficient target hybridisation, serum stability and the retention of antisense activity are key requirements for a successful artificial backbone (Figure 2B) [1]. As such, the PS modification is fully compatible with two of the predominant antisense approaches: (i) splice-switching to modulate mRNA maturation, or (ii) activation of RNase H to degrade an mRNA [38–40]. Moreover, advanced synthetic protocols [41] and favourable pharmacokinetics [42] of phosphorothioate oligonucleotides all contribute to its prevalence in clinically approved TherONs. However, PS backbones are linked to toxicity [43,44] and exploration of alternative modifications is urgently needed. A recent study showed that toxicity of PS-TherONs can be significantly reduced by a single MP substitution [45]. Other promising modalities of TherONs include peptide [46] and morpholino [47] nucleic acids which combine backbone and sugar modifications: The former combines amide bonds to connect acyclic subunits and the latter combines a phosphorodiamidate backbone and a morpholine ring as a sugar substitute. However, peptide nucleic acids suffer from poor solubility and inefficient cellular uptake [48,49] while morpholino oligonucleotides are associated with concerns for off-target effects [50,51]. Unfortunately, alternative backbones such as triazoles [32,33,52] or carbamates [53,54] reduce RNA target affinity which must be compensated for by additional sugar or base modifications [35,36,55,56]. Lengthy synthetic procedures limit the combination of artificial backbones with other base or sugar modifications. Nevertheless, these studies showcase the vast potential of alternative backbones in TherONs and emphasise the need for easily accessible backbone modifications for therapeutic research beyond specialised synthetic laboratories.

## Synthetic biology

### Nucleic acid formation

Different from short TherONs, modified long oligonucleotides for synthetic biology can be several hundreds of bases in length. This exceeds the limits of solid-phase oligonucleotide synthesis and requires different strategies. One approach is to assemble long modified oligomers from shorter, chemically modified ONs via ligation reactions (Figure 2C). Such ligation chemistry must be orthogonal to other functional groups within the oligomers and is often facilitated by a splint/template (orthogonal ligation, Figure 2C). A combinatorial approach for the discovery of splint-templated chemical ligations has been reported to identify DNA-compatible reactions to ligate terminally functionalised ONs [57]. Moreover, the generation of artificial backbone mimics has been shown for bridging 5′-*S*-phosphorothioester linkages (Ps) [58], PA [59–62], AM [61], urea [63], SQAM [63], TL1 [64] and TL3 [61]. Indeed, copper-catalysed azide-alkyne cycloaddition (CuAAC) to form TL3 was reported for the assembly of whole genes [12,65] and long RNA [9,10] from azide and alkyne modified shorter ONs. This approach enables the precise, site-specific introduction of artificial backbones and other modifications, but is limited by the compatibility of the ligation reaction with terminally modified ONs under aqueous conditions. Another approach is the introduction of artificial backbones through enzymatic synthesis using modified nucleotide triphosphates as substrates [4,13,66–68]. This has been demonstrated by the enzymatic synthesis of a PA-modified gene in the presence of an unnatural cytidine triphosphate analogue [13]. However, the controlled introduction of artificial backbones or other modifications at specific sites is not readily achievable with this method, and engineered polymerases are often required. Whilst engineered polymerases and ligases can accept base- and sugar-modified triphosphates as substrates, such incorporations still form PO bonds [69]. The engineering of enzymes to generate unnatural internucleoside linkages is inherently harder but has been recently demonstrated for uncharged ethylphosphonates [4].

### Biocompatibility of artificial backbones

Not all backbone-modified ONs have the desired biocompatibility for applications in synthetic biology. For instance, TL1 was recently described for the preparation of next-generation sequencing libraries but suffers from inefficient and inaccurate replication when used with several polymerases (polymerase read-through, Figure 2D) [34,64]. In contrast, TL3 has good read-through compatibility with DNA and RNA polymerases [70,71] and can be replicated with high fidelity [34] enabling expression of click-assembled genes in bacteria [12,59,70] and mammalian cells [11]. Similarly, phosphoramidate backbones can be read by DNA and RNA polymerases [59,61], and translated by ribosomes [60], and introduction of PA-modified genes can lead to the expression of their associated genetic information in bacteria [13]. Apart from gene synthesis, artificial backbones such as TL2, [31] urea [63] and SQAM [63] were reported as components of modified primers in PCR. In the case of SQAM, *in situ* template assembly by target-templated SQAM formation was utilised for RNA detection [63]. Other examples include the construction of a functional hammerhead ribozyme [9] or bioactive single guide RNAs (sgRNAs) for gene editing using a split-and-click strategy to form TL3 (CRISPR–Cas9 activation, Figure 2D) [10]. The versatility of artificial backbones in synthetic biology and biotechnology emphasises their vast potential. However, not all artificial backbones perform well, and the molecular requirements for biological integrity remain elusive [34]. Hence, easily accessible and structurally diverse artificial backbones are needed to fully exploit the vast potential of artificial nucleic acids in synthetic biology and biotechnology.

## Conclusion

Artificial backbones only account for a fraction of ON modifications but hold great potential. Despite many trailblazing discoveries emphasising the beneficial effects of artificial backbones in therapeutics and synthetic biology, their broad application and an in-depth understanding of their molecular requirements are hampered by limited synthetic accessibility. Thus, new chemical approaches are urgently needed for the synthesis of easy-to-access modified ONs to facilitate research on artificial backbones in a broader spectrum of laboratories.

## Open Access

Open access for this article was enabled by the participation of University of Oxford in an all-inclusive *Read & Publish* pilot with Portland Press and the Biochemical Society under a transformative agreement with JISC.

## Abbreviations

AM: amides
MP: methylphosphonates
ONs: Oligonucleotides
PA: phosphoramidates
PO: phosphodiester
PS: phosphorothioates
SQAM: squaramides
TherONs: therapeutic oligonucleotides
